# An ecological study on the association between universal health service coverage index, health expenditures, and early childhood caries

**DOI:** 10.1186/s12903-021-01500-8

**Published:** 2021-03-17

**Authors:** Morenike Oluwatoyin Folayan, Maha El Tantawi, Jorma I. Virtanen, Carlos Alberto Feldens, Maher Rashwan, Arthur M. Kemoli, Rita Villena, Ola B. Al-Batayneh, Rosa Amalia, Balgis Gaffar, Simin Z. Mohebbi, Arheiam Arheiam, Hamideh Daryanavard, Ana Vukovic, Robert J. Schroth, for the Early Childhood Caries Advocacy Group

**Affiliations:** 1 Department of Child Dental Health, Obafemi Awolowo University, Ile-Ife, Nigeria; 2 Alexandria University, Alexandria, Egypt; 3 Department of Clinical Dentistry, University of Bergen, Bergen, Norway; 4 Department of Pediatric Dentistry, Univesidade Luterana Do Brasil, Canoas, Brazil; 5 Centre for Oral Bioengineering, Barts and the London, School of Medicine and Dentistry, Queen Mary University of London, Mile End Road, London, E1 4NS UK; 6 Department of Conservative Dentistry, Faculty of Dentistry, Alexandria University, Alexandria, Egypt; 7 Department of Paediatric Dentistry and Orthodontics, University of Nairobi, Nairobi, Kenya; 8 Department of Pediatric Dentistry, San Martin de Porres University, Lima, Peru; 9 Department of Preventive Dentistry, Faculty of Dentistry, Jordan University of Science and Technology, Irbid, Jordan; 10 Preventive and Community Dentistry Department, Faculty of Dentistry, Universitas Gadjah Mada Yogyakarta, Yogyakarta, Indonesia; 11 Preventive Dental Sciences Department, College of Dentistry, Imam Abdulrahman Bin Faisal University, Dammam, Saudi Arabia; 12 Research Center for Caries Prevention, Dentistry Research Institute, Tehran University of Medical Sciences, Tehran, Iran; 13 Department of Community Oral Health, School of Dentistry, Tehran University of Medical Sciences, Tehran, Iran; 14 Department of Community and Preventive Dentistry, University of Benghazi, Benghazi, Libya; 15 Dubai Health Authority, Dubai, United Arab Emirates; 16 Department of Pediatric and Preventive Dentistry, School of Dental Medicine, University of Belgrade, Belgrade, Serbia; 17 Department of Preventive Dental Science, Dr. Gerald Niznick College of Dentistry, and Departments of Pediatrics and Child Health and Community Health Sciences, Max Rady College of Medicine, Rady Faculty of Health Sciences, University of Manitoba, Winnipeg, Canada

**Keywords:** Universal health coverage, Early childhood caries, Health expenditure

## Abstract

**Background:**

Universal health care (UHC) may assist families whose children are most prone to early childhood caries (ECC) in accessing dental treatment and prevention. The purpose of this study was to determine the association between UHC, health expenditure and the global prevalence of ECC.

**Methods:**

Health expenditure as percentage of gross domestic product, UHC service coverage index, and the percentage of 3–5-year-old children with ECC were compared among countries with various income levels using one-way analysis of variance (ANOVA). Three linear regression models were developed, and each was adjusted for the country income level with the prevalence of ECC in 3–5-year-old children being the dependent variable. In model 1, UHC service coverage index was the independent variable whereas in model 2, the independent variable was the health expenditure as percentage of GDP. Model 3 included both independent variables together. Regression coefficients (B), 95% confidence intervals (CIs), *P* values, and partial eta squared (ƞ^2^) as measure of effect size were calculated.

**Results:**

Linear regression including both independent factors revealed that health expenditure as percentage of GDP (*P* < 0.0001) was significantly associated with the percentage of ECC in 3–5-year-old children while UHC service coverage index was not significantly associated with the prevalence of ECC (*P* = 0.05). Every 1% increase in GDP allocated to health expenditure was associated with a 3.7% lower percentage of children with ECC (B = − 3.71, 95% CI: − 5.51, − 1.91). UHC service coverage index was not associated with the percentage of children with ECC (B = 0.61, 95% CI: − 0.01, 1.23). The impact of health expenditure on the prevalence of ECC was stronger than that of UHC coverage on the prevalence of ECC (ƞ^2^ = 0.18 vs. 0.05).

**Conclusions:**

Higher expenditure on health care may be associated with lower prevalence of ECC and may be a more viable approach to reducing early childhood oral health disparities than UHC alone. The findings suggest that currently, UHC is weakly associated with lower global prevalence of ECC.

**Supplementary Information:**

The online version contains supplementary material available at 10.1186/s12903-021-01500-8.

## Background

Early childhood caries (ECC) is defined as any caries experience involving the primary dentition in children less than 72 months of age [1–3]. ECC is one of the most prevalent chronic disease in early childhood [4], a risk factor for malnutrition [5–7] and a factor limiting growth, development and the quality of life of children with untreated lesions [8]. Its risk factors include individual (diet, oral hygiene, fluoride exposure, developmental enamel defects), family (household income, maternal level of education, and oral health knowledge, attitude, practice and psychosocial status) and community (water fluoridation, culture, environment and societal values) level factors [8–12]. There is ample literature on how individual and family factors are associated with ECC; but fewer and inconclusive studies assessing how country-level factors (macro-economic conditions, public policies, investment in health systems, public health funding) are associated with ECC [13–15].

El Tantawi et al. [16] recently reported that universal health coverage (UHC) may be a global risk indicator for ECC as the prevalence of ECC in children 3–5-years-old was lower in countries with good UHC. One explanation for this association was that countries with UHC may also promote improved access to preventive oral health care for preschool children. Investments in health through health expenditures can directly improve the country’s economy through maintaining the health of individuals and increasing their life expectancy [17].

The conceptualization of health as a fundamental human right [18] gave rise to the push for access to UHC which may enable the vulnerable members of the community who are most prone to ECC, to access preventive care and treatment of ECC [19]. However, UHC does not imply access to oral health care as not all countries that provide UHC coverages include oral health care in the health packages [19]. Among those countries that have integrated oral health care elements into UHC, many exclude preventive oral health care as this is considered a non-essential service [20]. Other reasons for its exclusion are low prioritization of oral health in many countries, the belief that oral health is an individual responsibility rather than social responsibility, and the high cost of dental services [21]. It is, therefore, important to assess not only how UHC may affect the risk of ECC but also to determine if countries’ health expenditure is associated with ECC. We postulate that health expenditures may be associated with family income as the lower a government’s health expenditures, the higher the out-of-pocket expenditures on health by families. When the costs of oral health care are high, families with limited financial means face additional financial stress because they cannot afford care [20]. Conversely, when government health expenditure is high, it is believed that children’s health and wellbeing improve, and children are protected against diseases that otherwise increase the risk of ECC [22].

Understanding how country-level policies and programs influence the risk of ECC is important for planning and policy decision-making geared towards prevention, diagnosis and treatment to limit severe sequels that may lead to poor later-life development. In addition, this enables ECC control and possible elimination where feasible [23]. This study assessed if there is an association between UHC service coverage, countries’ health expenditures and the prevalence of ECC. We hypothesize that the prevalence of ECC will have an inverse relationship with UHC and health spending.

## Methods

This was an ecological study based on data for UHC service coverage, health expenditure as percentage of gross domestic product (GDP), and the global prevalence of ECC in 3–5-year-old children. Additional file 1: Table S1 shows the values of the variables used for this study. These were:

### Early childhood caries (ECC)

Data on ECC prevalence were extracted from the World Health Organization Country Oral Health Profile database and studies published and indexed in MEDLINE, Scopus, Web of Science and Google Scholar covering the period 2007 to 2017. No language filter was applied. The retrieved data were used to calculate the ECC prevalence for each country by dividing the number of children affected by ECC in each study by the number of children examined and multiplying by 100. In the present study, we used the prevalence of ECC for 3–5-year-old children. Further details were reported in our previous paper [16].

### Universal health coverage (UHC)

Universal health service coverage index data were obtained from the World Bank Data Bank [24]. The index reflects the extent to which people receive healthcare services they need. Data used to calculate the index were obtained from responses to international surveys such as the Demographic Health Survey and the Multiple Indicator Cluster Survey. It represents coverage for essential health services (based on tracer interventions that include reproductive, maternal, newborn and child health, infectious diseases, noncommunicable diseases and service capacity and access). It is presented on a scale of 0 to 100 with higher values indicating greater coverage. The data for 2017 were used for this study.

### Health expenditure

It indicates health expenditure as percentage of GDP obtained from the World Bank Databank [25]. Estimates of the current health expenditures include healthcare goods and services consumed per year. This indicator does not include capital health expenditures such as buildings, machinery, information technology and stocks of vaccines for emergency or outbreaks. Data for 2017 were used to calculate this indicator.

### Confounders

Country income level is associated with ECC [16], universal health coverage [26] and total health expenditure [27]. We adjusted for country income level based on the 2017 Gross National Income (GNI) per capita calculated using the World Bank Atlas method [28]. Countries were grouped as: low-income (LICs-GDP of $995 or less); lower-middle-income (LMICs—GDP of $996–3895); upper-middle-income (UMICs-GDP of $3896–12,055); and high-income (HICs—GDP of $12,056 or more).

### Statistical analysis

Total health expenditure, UHC service coverage index, and the percentage of 3- to 5-year-old children with ECC were compared among countries with various income levels using one-way analysis of variance (ANOVA). Scheffe test was then used for post-hoc pairwise comparison. Three linear regression models were developed, and each was adjusted for the confounder (country income level) with the prevalence of ECC in 3–5-year-old children being the dependent variable. To control for the confounder, country income level was forced into each one of the three models so that the estimates produced are adjusted for it in the resulting multivariable models. In model 1, UHC service coverage index was the independent variable whereas in model 2, the independent variable was the health expenditure as percentage of GDP. Model 3 include both independent variables together. Thus, no stepwise selection was used. Based on a conceptual model, all variables were included in the models regardless of their *P* value. Regression coefficients (B), 95% confidence intervals (CIs), *P* values, and partial eta squared (ƞ^2^) as measure of effect size were calculated. Statistical analysis was conducted by SPSS version 22 (IBM Corp., Armonk, N.Y., USA). Significance was set at *P* < 0.05.

## Results

Combined data for the prevalence of ECC in 3–5-year-olds, UHC service coverage and the health expenditure was available for 83 countries (see Additional file 1: Table S1). The distribution of countries by income-level was as follows: LICs (6 countries), LMICs (20 countries), UMICs (24 countries), and HICs (33 countries).

Table 1 reports the UHC service coverage, health expenditure and percentage of ECC in 3–5-year-old children by income-levels. The percentage of 3–5-year-old children with ECC was significantly (*P* = 0.001) lower among HICs (45%) than LICs (63.1%), LMICs (64.8%), and UMICs (65.3%). Also, the UHC service coverage index score significantly increased (*P* < 0.0001) from LICS (44.3%) to LMICs (59.7%), UMICs (71.5%), and HICs (80.5%). Similarly, the health expenditure percentage of GDP significantly increased (*P* < 0.0001) from LICs (4.5%) to LMICs (4.7%), UMICs (6.2%) and HICs (8.5%).

**Table 1:** UHC service coverage, total per capita health expenditure and prevalence of ECC in 3–5-year-old children by income-levels

Variables	LICs	LMICs	UMICs	HICs	All	P
	6	20	24	33	83	
UHC service coverage index	44.33 (2.34) a	59.70 (10.10) b	71.50 (6.01) c	80.48 (5.50) d	70.27 (12.84)	< 0.0001*
Total health expenditure percentage of GDP	4.46 (1.15) a	4.65 (1.40) a	6.19 (2.18) a	8.48 (3.03) b	6.61 (2.86)	< 0.0001*
Percentage of 3–5-year-old children with ECC	63.12 (20.33) ab	64.77 (18.18) a	65.33 (17.54) a	44.97 (22.87) b	56.94 (22.10)	0.001*

*LIC* low-income country, *LMIC* lower middle-income country, *UMIC* upper middle-income country, *HIC* high income country

*Statistically significant at *P* ≤ 0.001, a, b, c, d: different letters denote columns that are statistically significant different from each other in same row. Same letters connote columns that are not statistically significantly different from each other in the same row

Table 2 reports the various regression models of the association between the percentage of 3–5-year-old children with ECC, UHC service coverage index, and health expenditure as a percentage of GDP. Model 1 revealed that there was no statistically significant association between the percentage of children with ECC and UHC service coverage index (*P* = 0.41). Model 1 explained 16% of the variation among countries in the percentage of children with ECC.

**Table 2:** Association between percentage of 3–5-year-old children with ECC and health expenditure and UHC

Variables	Model 1			Model 2			Model 3		
	B (95% CI)	*P* value	ƞ^2^	B (95% CI)	*P* value	ƞ^2^	B (95% CI)	*P* value	ƞ^2^
UHC service coverage index	0.27 (− 0.38, 0.93)	0.41	0.009	–			0.61 (− 0.01, 1.23)	0.05	0.05
Health expenditure % of GDP	–			− 3.25 (− 5.02, − 1.48)	< 0.0001*	0.15	− 3.71 (− 5.51; − 1.91)	< 0.0001*	0.18
Adjusted R2	0.16			0.28			0.30		

All models were adjusted for country income level

*B* regression coefficient, *CI* confidence interval, *Ƞ* partial eta squared

*Statistically significant at *P* < 0.05

Model 2 revealed that health expenditure as a percentage of GDP was significantly associated with the percentage of 3–5-year-old children with ECC (*P* < 0.0001); every 1% increase in the percentage of GDP allocated to health expenditure was associated with a reduction of 3.25% of the percentage of children with ECC. This model explained 28% of the variation in the percentage of children with ECC among countries.

Lastly, Model 3 included both independent factors and revealed that health expenditures as percentage of GDP was significantly associated with the percentage of 3–5-year-old children with ECC (*P* < 0.0001) while UHC service coverage index was not significantly associated with the percentage of 3–5-year-old children with ECC (*P* = 0.05). Every 1% increase in GDP allocated to health expenditure was associated with a 3.7% lower percentage of 3–5-year-old children with ECC.

(B = − 3.71, 95% CI: − 5.51, − 1.91). Meanwhile, UHC service coverage index was not associated with the percentage of 3–5-year-old children with ECC (B = 0.61, 95% CI: − 0.01, 1.23). The impact of health expenditure on the prevalence of ECC was stronger than that of UHC coverage (ƞ^2^ = 0.18 vs. 0.05). Overall, Model 3 explained 30% of the variation among countries in the percentage of 3–5-year-old children with ECC.

## Discussion

The findings from this study suggest that the greater expenditures on health care may be a protective factor for ECC, as the prevalence of ECC was lower in countries where expenditure on health was higher. UHC coverage was not significantly associated with the prevalence of ECC though the level of significance was at the threshold in the combined linear regression model. The study hypothesis was, therefore, partially sustained.

This global study is the first attempt to investigate empirically the possible relationship between countries’ expenditures on health care, UHC and ECC. Although ecological studies are useful for international data comparison and to generate study hypothesis, and this design is appropriate for the study we conducted, the findings of the present study should be interpreted with caution because of several limitations. These limitations include the potential for ecological fallacy, including using different datasets for information on the study variables [29]. We attempted to limit the disparity in the time when information on the study variables were collected by using an upper time boundary of 2017. Our study is also cross-sectional in design meaning that the findings do not suggest a cause-effect relationship. We were also unable to adjust for several recognized individual level risk factors for ECC, such as dental care utilization, use of fluoridated toothpaste, children’s oral-hygiene status, and the frequency of consumption of refined carbohydrates since no country-level data are available for these factors. Similarly, we also were unable to adjust for family level factors such as maternal oral health knowledge, attitude, practice and psychosocial status as there were no country-level data for these variables. However, we included country income level as confounder, thereby providing information that is important for the planning of regional and country-level interventions on oral health.

Studies have indicated that access to UHC and quality essential healthcare services with safe, effective, and affordable essential medicines and vaccines also improve oral health care and ensure timely access to caries prevention services [30, 31]. This study was unable to demonstrate a significant association between UHC and the prevalence of ECC in 3–5-year-old children howbeit the level of significance was at the threshold. This finding should be interpreted with caution as access to UHC for children does not directly translate to access to dental care regardless of whether UHC includes oral health care or not. Many countries do not have comprehensive oral health care included in their UHC programs [19, 32]. Additionally, many countries’ oral health care access programs fall short of addressing the needs of preschool children [33].

UHC packages are dependent on the country’s economy, health plans, social, environmental and political determinants of health [34]. Studies conducted in Canada and the UK reveal that access to dental insurance programs for children did not eliminate the disparities in caries experience [35, 36]. Also, in Brazil, though access to UHC significantly reduced infant mortality rate, the prevalence of ECC in children up to 5 years of age was still very high [37]. In Peru, access to UHC had no impact on the oral health of children under 12 years of age [38]. However, studies in South Africa and Nordic countries reported that access to free oral health care reduced caries severity in children [39, 40].

UHC may not improve dental service utilization for preventive oral health care [41] especially in developing countries were the perceived need for dental care is low even though the rate of unmet dental care needs is relatively high [42]. In addition, access to oral health care as measured by dental visits had limited impact for those most vulnerable to ECC [43] despite advocacy for early child visits to the dental clinic [44]. This study suggests that UHC and access to oral health care may be different constructs.

ECC results from a complex interaction between biological, behavioral and structural risk factors that may not be eliminated by UHC. Achieving UHC depends on quality health service delivery systems with adequate and appropriate personnel and health facilities in addition to effective health communication and information systems, governance, evaluation and monitoring of the quality of services provided [45]. The results of the present study may indicate that in many countries, the impact of UHC may be diluted by challenges to be overcome in these complex interactions, especially in LMICs. Future studies need to assess the impact of UHC, with and without oral health care components, on the prevalence of ECC; and how access to UHC can be optimized to reduce the risk to ECC.

The study showed that as expenditure on health increases, the prevalence of ECC decreases. This may imply that increasing government’s expenditure on health increases the oral health care service provision coverage and thereby improve oral health care availability, and accessibility which UHC coverage may not be able to address. Governments’ investment in oral health care is nevertheless critical as this affects the equitable distribution of the needed infrastructure to provide oral health care, as well as the distribution of human resources needed to ensure access to quality oral health preventive and curative care [16]. The study finding agrees with Markovic et al. [46] who demonstrated an association between health expenditure and ECC. Advocacy for the inclusion of comprehensive oral health care in UHC packages should be accompanied by increased governmental spending on health. There are multiple global, regional and national political instruments that can support the advocacy for increased resourcing for healthcare [47, 48]. In addition, there are many organizations supporting UHC in low-, middle- and high-income countries [49]. Providing preliminary evidence on the impact of health expenditure on the oral health of children will further strengthen the case for health financing as an instrument to improve the overall health of children.

## Conclusion

Higher health expenditure may be associated with lower prevalence of ECC. Evidence suggests that currently, UHC may not be significantly associated with lower global prevalence of ECC. Further country specific studies are needed to understand how UHC may reduce the risk of ECC; and complement the evidence this study provides on health expenditure association with lower prevalence of ECC globally.

## Supplementary Information

**Additional file 1:** The global prevalence of early childhood caries, universal health coverage service coverage index, and total health expenditure per capita.

## Data Availability

Study related materials are public data. All study related data are included in the supplemental file of this manuscript.

## Abbreviations

B: Régression coefficient
CI: Confidence interval
ECC: Early Childhood Caries
GDP: Gross domestic product
HICs: High-income countries
LICs: Low-income countries
LMICs: Low middle income countries
ƞ^2^: Partial eta squared
UHC: Universal health coverage
UMICs: Upper middle-income countries
