# Correction to: An ecological study on the association between universal health service coverage index, health expenditures, and early childhood caries

**DOI:** 10.1186/s12903-021-01624-x

**Published:** 2021-05-26

**Authors:** Morenike Oluwatoyin Folayan, Maha El Tantawi, Jorma I. Virtanen, Carlos Alberto Feldens, Maher Rashwan, Arthur M. Kemoli, Rita Villena, Ola B. Al-Batayneh, Rosa Amalia, Balgis Gafar, Simin Z. Mohebbi, Arheiam Arheiam, Hamideh Daryanavard, Ana Vukovic, Robert J. Schroth, for the Early Childhood Caries Advocacy Group

**Affiliations:** 1 Department of Child Dental Health, Obafemi Awolowo University, Ile-Ife, Nigeria; 2 Alexandria University, Alexandria, Egypt; 3 Department of Clinical Dentistry, University of Bergen, Bergen, Norway; 4 Department of Pediatric Dentistry, Univesidade Luterana do Brasil, Canoas, Brazil; 5 Centre for Oral Bioengineering, Barts and the London, School of Medicine and Dentistry, Queen Mary University of London, Mile End Road, London, E1 4NS UK; 6 Department of Conservative Dentistry, Faculty of Dentistry, Alexandria University, Alexandria, Egypt; 7 Department of Paediatric Dentistry and Orthodontics, University of Nairobi, Nairobi, Kenya; 8 Department of Pediatric Dentistry, San Martin de Porres University, Lima, Peru; 9 Department of Preventive Dentistry, Faculty of Dentistry, Jordan University of Science and Technology, Irbid, Jordan; 10 Preventive and Community Dentistry Department, Faculty of Dentistry, Universitas Gadjah Mada Yogyakarta, Yogyakarta, Indonesia; 11 Preventive Dental Sciences Department, College of Dentistry, Imam Abdulrahman Bin Faisal University, Dammam, Saudi Arabia; 12 Department of Community Oral Health, School of Dentistry, Tehran University of Medical Sciences, Tehran, Iran; 13 Department of Community and Preventive Dentistry, University of Benghazi, Benghazi, Libya; 14 Dubai Health Authority, Dubai, United Arab Emirates; 15 Department of Pediatric and Preventive Dentistry, School of Dental Medicine, University of Belgrade, Belgrade, Serbia; 16 Department of Preventive Dental Science, Dr. Gerald Niznick College of Dentistry, and Departments of Pediatrics and Child Health and Community Health Sciences, Max Rady College of Medicine, Rady Faculty of Health Sciences, University of Manitoba, Winnipeg, Canada

## Correction to: BMC Oral Health (2021) 21:126 https://doi.org/10.1186/s12903-021-01500-8

After publication of the original article [1], the authors identified an error in Dr. Balgis Gaffar’s affiliations list.

The incorrect author’s affiliations list is:

11 Preventive Dental Sciences Department, College of Dentistry, Imam Abdulrahman Bin Faisal University, Dammam, Saudi Arabia.

12 Research Center for Caries Prevention, Dentistry Research Institute, Tehran University of Medical Sciences, Tehran, Iran.

The correct author’s affiliations list is:

Preventive Dental Sciences Department, College of Dentistry, Imam Abdulrahman Bin Faisal University, Dammam, Saudi Arabia.

The original article has been corrected.
