# Supplementary material for: An ecological study on the association between universal health service coverage index, health expenditures, and early childhood caries
Source: BMC Oral Health. 2021 Mar 17;21:126. doi: 10.1186/s12903-021-01500-8 (PMC7968322; doi:10.1186/s12903-021-01500-8)
Supplement: Supplementary file 1 — Additional file 1: The global prevalence of early childhood caries, universal health coverage service coverage index, and total health expenditure per capita. [file 12903_2021_1500_MOESM1_ESM.doc]

|  | | **Table 1: UNIVERSAL EFFECTVE HEALTH COVERAG INDEX, UNDER-5 SERVICE UNIVERSAL HEALTH AND EARLY CHILDHOOD CARIES PREVALENCE BY COUNTRY** | | | | | | | | |
| --- | --- | --- | --- | --- | --- | --- | --- | --- | --- | --- |
| **ID** | COUNTRY | | ECC PREVALENCE IN CHILDREN AGED <36 MONTHS | ECC PREVALENCE IN CHILDREN AGED 36-71 MONTHS | UNIVERSAL HEALTH COVERAGE INDEX  2017 | UNIVERSAL EFFECTIVE HEALTH COVERAGE INDEX | DIPHTHERIA-TETANUS-PERTUSSIS VACCINE, 3 DOSE SERVICE COVERAGE | MEASLES CONTAINING VACCINE, 1 DOSE SERVICE COVERAGE | LOWER RESPIRATORY INFECTIONS TREATMENT BASED ON MORTALITY-TO-INCIDENCE RATIO SERVICE COVERAGE | DIARRHOEAL DISEASES TREATMENT BASED ON MORTALITY-TO-INCIDENCE RATIO SERVICE COVERAGE |
| 1 | Afghanistan | | No data | No data | 37.00 | 46 | 62 | 65 | 70 | 91 |
| 2 | Albania | | No data | 91.0 | 59.00 | 74 | 93 | 98 | 84 | 100 |
| 3 | Algeria | | No data | No data | 78.00 | 69 | 84 | 92 | 96 | 99 |
|  | American Samoa | | No data | No data | No data | 54 | 89 | 85 | 95 | 99 |
| 4 | Andorra | | No data | No data | No data | 83 | 98 | 99 | 100 | 100 |
| 5 | Angola | | No data | No data | 40.00 | 46 | 53 | 42 | 69 | 80 |
| 6 | Antigua & Barbuda | | No data | Data before 2007 | 73.00 | 65 | 94 | 93 | 93 | 99 |
| 7 | Argentina | | Data before 2007 | 80.4 | 76.00 | 70 | 93 | 86 | 97 | 99 |
| 8 | Armenia | | No data | No data | 69.00 | 68 | 94 | 93 | 71 | 99 |
|  | Aruba | | No data | No data | No data | No data | No data | No data | No data | No data |
| 9 | Australia | | 23.0 | 49.8 | 87.00 | 92 | 91 | 96 | 100 | 100 |
| 10 | Austria | | No data | 42.6 | 79.00 | 89 | 95 | 88 | 100 | 100 |
| 11 | Azerbaijan | | No data | No data | 65.00 | 50 | 87 | 82 | 17 | 97 |
| 12 | Bahamas | | No data | Data before 2007 | 75.00 | 63 | 88 | 91 | 92 | 99 |
| 13 | Bahrain | | No data | No data | 77.00 | 79 | 99 | 100 | 100 | 100 |
| 14 | Bangladesh | | No data | No data | 48.00 | 62 | 94 | 92 | 84 | 96 |
| 15 | Barbados | | No data | No data | 77.00 | 68 | 92 | 92 | 96 | 100 |
| 16 | Belarus | | 28.1 | No data | 76.00 | 67 | 97 | 100 | 99 | 100 |
| 17 | Belgium | | 9.6 | 30.0 | 84.00 | 88 | 96 | 99 | 100 | 100 |
| 18 | Belize | | No data | No data | 64.00 | 58 | 92 | 82 | 87 | 97 |
| 19 | Benin | | No data | No data | 40.00 | 51 | 73 | 80 | 45 | 75 |
|  | Bermuda | | No data | No data | No data | 82 | 99 | 99 | 99 | 100 |
| 20 | Bhutan | | No data | No data | 62.00 | 62 | 99 | 100 | 87 | 96 |
| 21 | Bolivia | | No data | No data | 68.00 | No data | No data | No data | No data | No data |
| 22 | Bosnia & Herzegovina | | No data | 83.0 | 61.00 | No data | No data | No data | No data | No data |
| 23 | Botswana | | No data | No data | 61.00 | 63 | 86 | 84 | 73 | 83 |
| 24 | Brazil | | 20.3 | 51.3 | 79.00 | 70 | 96 | 86 | 88 | 98 |
|  | British Virgin | | No data | No data | No data | No data | No data | No data | No data | No data |
| 25 | Brunei | | No data | 59.0 | 81.00 | 65 | 98 | 98 | 98 | 100 |
| 26 | Bulgaria | | Data before 2007 | No data | 66.00 | 64 | 92 | 91 | 89 | 99 |
| 27 | Burkina Faso | | No data | No data | 40.00 | 50 | 95 | 94 | 6 | 67 |
| 28 | Burundi | | No data | No data | 42.00 | 56 | 93 | 95 | 76 | 66 |
| 29 | Cambodia | | Data before 2007 | 78.8 | 60.00 | 62 | 91 | 92 | 66 | 97 |
| 30 | Cameroon | | No data | No data | 46.00 | 46 | 72 | 76 | 48 | 59 |
| 31 | Canada | | 36.2 | 85.6 | 89.00 | 91 | 93 | 86 | 100 | 100 |
| 32 | Cape Verde | | No data | No data | 69.00 | 67 | 100 | 100 | 87 | 97 |
|  | Caribbean | | No data | No data | No data | No data | No data | No data | No data | No data |
|  | Cayman island | | No data | No data | No data | No data | No data | No data | No data | No data |
| 33 | Central African Republic | | No data | No data | 33.00 | 28 | 49 | 48 | 37 | 17 |
| 34 | Chad | | No data | No data | 28.00 | 33 | 57 | 39 | 27 | 17 |
|  | Channel Island | | No data | No data | No data | No data | No data | No data | No data | No data |
| 35 | Chile | | 29.3 | 53.6 | 70.00 | 76 | 94 | 93 | 99 | 100 |
| 36 | China | | 8.8 | 62.2 | 79.00 | 76 | 99 | 99 | 94 | 100 |
|  | Christmas | | No data | No data | No data | No data | No data | No data | No data | No data |
|  | Cocos Island | | No data | No data | No data | No data | No data | No data | No data | No data |
| 37 | Colombia | | 19.2 | 75.3 | 76.00 | 81 | 95 | 91 | 94 | 99 |
| 38 | Comoros | | No data | No data | 52.00 | 51 | 83 | 84 | 68 | 85 |
| 39 | Congo | | No data | No data | 39.00 | 49 | 73 | 65 | 87 | 82 |
|  | Cook Island | | No data | No data | No data | 58 | 88 | 90 | 98 | 100 |
| 40 | Congo, DRC | | No data | 80.0 | 41.00 | 53 | 82 | 73 | 80 | 81 |
| 41 | Costa Rica | | No data | No data | 77.00 | 83 | 100 | 98 | 99 | 99 |
| 42 | Cote d'Ivoire | | No data | No data | 47.00 | 48 | 72 | 79 | 45 | 83 |
| 43 | Croatia | | No data | Data before 2007 | 71.00 | 82 | 90 | 91 | 100 | 100 |
| 44 | Cuba | | No data | No data | 83.00 | 77 | 98 | 99 | 98 | 100 |
|  | Curacao | | No data | No data | No data | No data | No data | No data | No data | No data |
| 45 | Cyprus | | No data | No data | 78.00 | 87 | 89 | 99 | 100 | 100 |
| 46 | Czech Republic | | No data | 55.1 | 76.00 | 85 | 95 | 95 | 99 | 100 |
| 47 | Denmark | | No data | 6.3 | 81.00 | 88 | 92 | 97 | 100 | 100 |
| 48 | Djibouti | | No data | No data | 47.00 | 48 | 83 | 57 | 64 | 88 |
| 49 | Dominica | | No data | No data |  | 58 | 86 | 94 | 86 | 98 |
| 50 | Dominican Republic | | No data | No data | 74.00 | 58 | 85 | 75 | 93 | 97 |
| 51 | Ecuador | | 26.0 | 44.8 | 77.00 | 66 | 77 | 82 | 88 | 99 |
| 52 | East Timor | | No data | No data | No data | No data | No data | No data | No data | No data |
| 53 | Egypt | | 69.6 | 61.6 | 68.00 | 59 | 95 | 97 | 89 | 89 |
| 54 | El Salvador | | 30.0 | 85.0 | 76.00 | 68 | 93 | 87 | 96 | 98 |
|  | England | | No data | No data | No data | No data | No data | No data | No data | No data |
| 55 | Equatorial Guinea | | No data | No data | 45.00 | 55 | 39 | 45 | 87 | 97 |
| 56 | Eritrea | | No data | No data | 38.00 | 46 | 100 | 95 | 73 | 72 |
| 57 | Estonia | | No data | 42.0 | 75.00 | 86 | 94 | 100 | 98 | 100 |
| 58 | Ethiopia | | No data | Data before 2007 | 39.00 | 55 | 60 | 57 | 69 | 69 |
|  | Faeroe Island | | No data | No data | No data | No data | No data | No data | No data | No data |
|  | Falkland Island | | No data | No data | No data | No data | No data | No data | No data | No data |
| 59 | Fiji | | Data before 2007 | No data | 64.00 | 44 | 80 | 85 | 87 | 94 |
| 60 | Finland | | 0.3 | 20.4 | 78.00 | 89 | 93 | 88 | 100 | 100 |
| 61 | France | | No data | 28.8 | 78.00 | 95 | 90 | 97 | 100 | 100 |
|  | French Polynesia | | No data | No data | No data | No data | No data | No data | No data | No data |
| 62 | Gabon | | No data | No data | 49.00 | 54 | 69 | 78 | 90 | 92 |
| 63 | Gambia | | No data | 86.0 | 44.00 | 54 | 93 | 88 | 77 | 91 |
| 64 | Georgia | | No data | 51.6 | 66.00 | 60 | 93 | 84 | 87 | 99 |
| 65 | Germany | | 18.0 | 28.2 | 83.00 | 88 | 97 | 97 | 100 | 100 |
| 66 | Ghana | | No data | Data before 2007 | 47.00 | 54 | 94 | 94 | 73 | 89 |
|  | Gilbratrar | | No data | No data | No data | No data | No data | No data | No data | No data |
| 67 | Greece | | 14.0 | 32.3 | 75.00 | 82 | 97 | 100 | 99 | 100 |
|  | Greenland | | No data | No data | No data | 67 | 92 | 94 | 98 | 99 |
| 68 | Grenada | | No data | No data | 72.00 | 54 | 92 | 89 | 90 | 100 |
|  | Guadeloupe | | No data | No data | No data | No data | No data | No data | No data | No data |
|  | Guam | | No data | No data | No data | 63 | 95 | 92 | 92 | 100 |
| 69 | Guatemala | | Data before 2007 | Data before 2007 | 55.00 | 61 | 87 | 78 | 80 | 92 |
| 70 | Guinea | | No data | Data before 2007 | 37.00 | 38 | 53 | 48 | 35 | 72 |
| 71 | Guinea-Bissau | | No data | No data | 40.00 | 47 | 81 | 74 | 71 | 63 |
| 72 | Guyana | | No data | No data | 72.00 | 41 | 95 | 91 | 86 | 94 |
| 73 | Haiti | | No data | No data | 49.00 | 40 | 74 | 77 | 63 | 72 |
| 74 | Honduras | | No data | Data before 2007 | 65.00 | 63 | 93 | 89 | 96 | 93 |
|  | Hong Kong | | No data | No data | No data | No data | No data | No data | No data | No data |
| 75 | Hungary | | No data | Data before 2007 | 74.00 | 74 | 98 | 98 | 99 | 100 |
| 76 | Iceland | | No data | Data before 2007 | 84.00 | 93 | 94 | 91 | 100 | 100 |
| 77 | India | | 38.9 | 52.1 | 55.00 | 58 | 94 | 89 | 67 | 81 |
| 78 | Indonesia | | 35.8 | 79.5 | 57.00 | 52 | 81 | 77 | 87 | 88 |
| 79 | Iran | | No data | 55.5 | 72.00 | 74 | 99 | 99 | 97 | 99 |
| 80 | Iraq | | No data | 82.0 | 61.00 | 65 | 79 | 78 | 95 | 99 |
| 81 | Ireland | | No data | 26.5 | 76.00 | 91 | 91 | 95 | 100 | 100 |
|  | Northern Ireland | | No data | No data | No data | 89 | 91 | 95 | 100 | 100 |
|  | Isle of Man | | No data | No data | No data | No data | No data | No data | No data | No data |
| 82 | Israel | | 17.6 | 64.7 | 82.00 | 89 | 98 | 96 | 100 | 100 |
| 83 | Italy | | 15.4 | 26.1 | 82.00 | 93 | 93 | 96 | 100 | 100 |
| 84 | Jamaica | | No data | 49.0 | 65.00 | 62 | 100 | 96 | 97 | 99 |
| 85 | Japan | | 3.9 | 23.9 | 83.00 | 95 | 97 | 98 | 100 | 100 |
|  | Jersey | | No data | No data | No data | No data | No data | No data | No data | No data |
| 86 | Jordan | | No data | Data before 2007 | 76.00 | 75 | 81 | 90 | 94 | 100 |
| 87 | Kazakhstan | | 45.0 | 70.0 | 76.00 | 60 | 95 | 95 | 74 | 99 |
| 88 | Kenya | | No data | 64.2 | 55.00 | 56 | 77 | 84 | 85 | 75 |
| 89 | Kiribati | | No data | No data | 41.00 | 38 | 80 | 70 | 88 | 90 |
| 90 | South Korea | | Data before 2007 | 82.0 | 71.00 | 55 | 99 | 100 | 97 | 97 |
| 91 | North Korea | | No data | No data | 86.00 | 93 |  |  |  |  |
| 92 | Kuwait | | 3.0 | 28.0 | 76.00 | 90 | 95 | 99 | 98 | 100 |
| 93 | Kyrgyzstan | | 45.4 | 69.8 | 70.00 | 59 | 91 | 92 | 62 | 96 |
| 94 | Laos | | No data | 80.5 | 51.00 | 50 | 70 | 62 | 50 | 89 |
| 95 | Latvia | | 26.0 | Data before 2007 | 71.00 | 71 | 100 | 100 | 98 | 100 |
| 96 | Lebanon | | No data | 74.7 | 73.00 | 80 | 81 | 76 | 99 | 100 |
| 97 | Lesotho | | No data | No data | 48.00 | 50 | 99 | 99 | 58 | 72 |
| 98 | Liberia | | No data | No data | 39.00 | 51 | 93 | 89 | 75 | 66 |
| 99 | Libya | | No data | 75.0 | 64.00 | 68 | 87 | 88 | 98 | 100 |
| 100 | Liechtenstein | | No data | No data | No data | No data | No data | No data | No data | No data |
| 101 | Lithuania | | No data | 71.8 | 73.00 | 70 | 90 | 100 | 97 | 100 |
| 102 | Luxembourg | | No data | No data | 83.00 | 94 | 99 | 100 | 100 | 100 |
|  | Macao | | No data | No data | No data | No data | No data | No data | No data | No data |
| 103 | Macedonia | | No data | No data | No data | No data | No data | No data | No data | No data |
| 104 | Madagascar | | No data | Data before 2007 | 28.00 | 44 | 74 | 86 | 69 | 59 |
| 105 | Malawi | | No data | No data | 46.00 | 64 | 93 | 92 | 67 | 85 |
| 106 | Malaysia | | No data | 98.1 | 73.00 | 68 | 90 | 90 | 99 | 100 |
| 107 | Maldives | | No data | No data | 62.00 | 70 | 91 | 87 | 97 | 99 |
| 108 | Mali | | No data | No data | 38.00 | 45 | 71 | 69 | 21 | 74 |
| 109 | Malta | | No data | No data | 82.00 | 87 | 94 | 96 | 99 | 100 |
| 110 | Marshall Is. | | No data | No data | No data | 45 | 88 | 64 | 86 | 98 |
|  | Martinique | | No data | No data | No data | No data | No data | No data | No data | No data |
| 111 | Mauritania | | No data | No data | 41.00 | 57 | 80 | 69 | 84 | 81 |
| 112 | Mauritius | | No data | No data | 63.00 | 54 | 85 | 85 | 97 | 99 |
|  | Mayotte | | No data | No data | No data | No data | No data | No data | No data | No data |
| 113 | Mexico | | 34.0 | 61.5 | 76.00 | 68 | 84 | 74 | 90 | 97 |
| 114 | Micronesia | | No data | No data | 47.00 | 36 | 66 | 65 | 89 | 98 |
| 115 | Moldova | | No data | No data | 69.00 | No data | No data | No data | No data | No data |
| 116 | Monaco | | No data | No data | No data | 89 | No data | No data | No data | No data |
| 117 | Mongolia | | 47.5 | 91.5 | 62.00 | 56 | 92 | 96 | 68 | 97 |
| 118 | Montenegro | | No data | No data | 68.00 | 73 | 61 | 87 | 99 | 100 |
|  | Montserrat | | No data | No data | No data | No data | No data | No data | No data | No data |
| 119 | Morocco | | 9.0 | 60.0 | 70.00 | 60 | 91 | 96 | 93 | 95 |
| 120 | Mozambique | | No data | No data | 46.00 | 53 | 97 | 95 | 56 | 84 |
| 121 | Myanmar | | Data before 2007 | 50.0 | 61.00 | 54 | 84 | 73 | 59 | 93 |
| 122 | Namibia | | 31.3 | 68.7 | 62.00 | 70 | 100 | 92 | 83 | 90 |
| 123 | Nauru | | No data | No data | No data | 44 | 100 | 99 | 79 | 98 |
| 124 | Nepal | | No data | 61.5 | 48.00 | 59 | 95 | 86 | 82 | 94 |
| 125 | Netherlands | | No data | 56.0 | 86.00 | 90 | 93 | 95 | 100 | 100 |
|  | New Caledonia | | No data | No data | No data | No data | No data | No data | No data | No data |
| 126 | New Zealand | | No data | 88.0 | 87.00 | 85 | 91 | 95 | 99 | 100 |
| 127 | Nicaragua | | No data | Data before 2007 | 73.00 | 68 | 100 | 100 | 90 | 97 |
| 128 | Niger | | No data | No data | 37.00 | 42 | 64 | 60 | 26 | 38 |
| 129 | Nigeria | | 2.7 | 14.9 | 42.00 | 43 | 55 | 51 | 1 | 33 |
|  | Niue | | No data | No data | No data | 50 | 89 | 89 | 85 | 99 |
|  | Northern Mariana | | No data | No data | No data | 60 | No data | No data | No data | No data |
| 130 | Norway | | No data | 22.5 | 87.00 | 89 | 97 | 97 | 100 | 100 |
|  | Oceania | | No data | No data | No data | No data | No data | No data | No data | No data |
| 131 | Oman | | No data | No data | 69.00 | 73 | 88 | 89 | 98 | 100 |
| 132 | Pakistan | | 26.5 | 60.0 | 45.00 | 46 | 74 | 80 | 63 | 68 |
| 133 | Palau | | No data | Data before 2007 | No data | 44 | 81 | 87 | 84 | 99 |
|  | Palestine | | No data | No data | No data | 67 | 99 | 99 | 99 | 100 |
| 134 | Panama | | No data | No data | 79.00 | 76 | 92 | 82 | 91 | 96 |
| 135 | Papua New Guinea | | No data | No data | 40.00 | 40 | 60 | 60 | 51 | 83 |
| 136 | Paraguay | | 10.2 | 81.0 | 69.00 | 65 | 93 | 92 | 94 | 98 |
| 137 | Peru | | No data | 76.0 | 77.00 | 79 | 83 | 76 | 93 | 98 |
| 138 | Philippines | | Data before 2007 | 93.0 | 61.00 | 55 | 85 | 81 | 81 | 95 |
|  | Pitcairn | | No data | No data | No data | No data | No data | No data | No data | No data |
| 139 | Poland | | No data | 52.9 | 75.00 | 78 | 93 | 95 | 98 | 100 |
| 140 | Portugal | | No data | No data | 82.00 | 87 | 99 | 98 | 99 | 100 |
|  | Puerto Rica | | No data | No data | No data | 80 | No data | No data | No data | No data |
| 141 | Qatar | | No data | 89.2 | 68.00 | 84 | 99 | 94 | 100 | 100 |
|  | Reunion | | No data | No data | No data | No data | No data | No data | No data | No data |
| 142 | Romania | | Data before 2007 | 81.1 | 74.00 | 67 | 88 | 86 | 80 | 100 |
| 143 | Russia | | 36.0 | 49.0 | 74.00 | 65 | 100 | 100 | 95 | 100 |
| 144 | Rwanda | | No data | No data | 57.00 | 66 | 96 | 97 | 76 | 86 |
|  | St Barthlemy | | No data | No data | No data | No data | No data | No data | No data | No data |
|  | St Helena | | No data | No data | No data | No data | No data | No data | No data | No data |
| 145 | St. Kitts & Nevis | | No data | No data | No data | 55 | 94 | 96 | 92 | 98 |
| 146 | St. Lucia | | No data | No data | No data | 62 | 88 | 88 | 95 | 99 |
|  | St Martin | | No data | No data | No data | No data | No data | No data | No data | No data |
|  | St Pierre and | | No data | No data | No data | No data | No data | No data | No data | No data |
| 147 | St. Vincent & the Grenadines | | No data | No data | No data | 55 | 98 | 94 | 93 | 98 |
| 148 | Samoa | | No data | No data | 58.00 | 53 | 53 | 52 | 93 | 99 |
| 149 | San Marino | | No data | No data |  | 95 | 87 | 89 | 100 | 100 |
| 150 | Sao Tome & Principe | | No data | No data | 55.00 | 51 | 94 | 94 | 79 | 98 |
| 151 | Saudi Arabia | | Data before 2007 | 70.1 | 74.00 | 64 | 99 | 98 | 100 | 100 |
|  | Scotland | | No data | No data | No data | No data | No data | No data | No data | No data |
| 152 | Senegal | | No data | 73.0 | 45.00 | 56 | 86 | 93 | 78 | 83 |
| 153 | Serbia | | 37.5 | 19.8 | 65.00 | 70 | 91 | 85 | 99 | 100 |
| 154 | Seychelles | | No data | No data | 71.00 | 62 | 90 | 89 | 92 | 99 |
| 155 | Sierra Leone | | No data | No data | 39.00 | 45 | 87 | 86 | 19 | 83 |
| 156 | Singapore | | No data | 49.0 | 86.00 | 93 | 95 | 93 | 100 | 100 |
|  | Sint Marteen | | No data | No data | No data | No data | No data | No data | No data | No data |
| 157 | Slovakia | | No data | No data | 77.00 | 79 | 94 | 95 | 95 | 100 |
| 158 | Slovenia | | No data | No data | 79.00 | 93 | 91 | 93 | 100 | 100 |
| 159 | Solomon Is. | | No data | No data | 47.00 | 47 | 98 | 86 | 83 | 93 |
| 160 | Somalia | | No data | No data | 25.00 | 28 | 57 | 31 | 33 | 63 |
| 161 | South Africa | | No data | 44.0 | 69.00 | 66 | 79 | 63 | 80 | 71 |
| 162 | Spain | | No data | 16.4 | 83.00 | 94 | 95 | 93 | 100 | 100 |
| 163 | Sri Lanka | | 24.5 | 57.7 | 66.00 | 72 | 97 | 96 | 98 | 100 |
| 164 | North Sudan | | No data | 58.5 | 44.00 | 61 | 85 | 78 | 90 | 90 |
| 165 | South Sudan | | No data | Data before 2007 | 31.00 | 49 | No data | No data | No data | No data |
| 166 | Suriname | | No data | No data | 71.00 | 54 | 89 | 99 | 86 | 95 |
| 167 | Swaziland | | No data | No data | 63.00 | 58 | 94 | 87 | 65 | 79 |
| 168 | Sweden | | 6.0 | 19.1 | 86.00 | 90 | 98 | 98 | 100 | 100 |
| 169 | Switzerland | | 25.3 | 24.8 | 83.00 | 96 | 95 | 98 | 100 | 100 |
| 170 | Syria | | No data | 65.8 | 60.00 | 61 | 76 | 68 | 96 | 99 |
|  | Taiwan | | No data | No data | No data | 85 | 100 | 98 | 100 | 100 |
| 171 | Tajikistan | | No data | No data | 68.00 | 53 | 96 | 93 | 19 | 87 |
| 172 | Tanzania | | 5.2 | 37.3 | 43.00 | 63 | 88 | 91 | 68 | 90 |
| 173 | Thailand | | No data | 67.2 | 80.00 | 75 | 92 | 89 | 97 | 99 |
|  | Timor -Leste | | No data | No data | 52.00 | 52 | 68 | 63 | 76 | 93 |
| 174 | Togo | | No data | No data | 43.00 | 46 | 84 | 86 | 67 | 49 |
|  | Tokelau | | No data | No data | No data | 55 | 90 | 86 | 95 | 99 |
| 175 | Tonga | | No data | No data | 58.00 | 57 | 67 | 68 | 92 | 100 |
| 176 | Trinidad & Tobago | | No data | 29.1 | 74.00 | 58 | 90 | 78 | 96 | 99 |
| 177 | Tunisia | | No data | No data | 70.00 | 71 | 94 | 96 | 98 | 100 |
| 178 | Turkey | | No data | 60.3 | 74.00 | 76 | 97 | 98 | 97 | 99 |
| 179 | Turkmenistan | | No data | No data | 70.00 | 48 | 94 | 100 | 19 | 95 |
|  | Turks and Caicos Island | | No data | No data | No data | No data | No data | No data | No data | No data |
| 180 | Tuvalu | | No data | No data |  | 40 | 67 | 63 | 89 | 99 |
| 181 | Uganda | | 17.8 | 41.0 | 45.00 | 63 | 80 | 79 | 85 | 90 |
| 182 | Ukraine | | 23.8 | 56.7 | 68.00 | 55 | 98 | 72 | 96 | 100 |
| 183 | United Arab Emirates | | Data before 2007 | 67.6 | 76.00 | 62 | 92 | 97 | 100 | 100 |
| 184 | United Kingdom | | Data before 2007 | 40.2 | 87.00 | 87 | 93 | 96 | 99 | 100 |
| 185 | United States | | 27.2 | 31.8 | 84.00 | 84 | 93 | 93 | 100 | 100 |
|  | United States Virgin | | No data | No data | No data | 60 | 65 | 61 | 99 | 100 |
| 186 | Uruguay | | No data | Data before 2007 | 80.00 | 73 | 97 | 90 | 98 | 99 |
| 187 | Uzbekistan | | No data | No data | 73.00 | 47 | 91 | 95 | 7 | 99 |
| 188 | Vanuatu | | No data | 42.1 | 48.00 | 36 | 45 | 51 | 82 | 95 |
|  | Vatican | | No data | No data | No data | No data | No data | No data | No data | No data |
| 189 | Venezuela | | Data before 2007 | 87.0 | 74.00 | 68 | 57 | 56 | 95 | 97 |
| 190 | Vietnam | | No data | 73.7 | 75.00 | 64 | 91 | 85 | 91 | 100 |
|  | Wales | | No data | No data | No data | No data | No data | No data | No data | No data |
|  | Wales and Funtana | | No data | No data | No data | No data | No data | No data | No data | No data |
|  | Western Sahara | | No data | No data | No data | No data | No data | No data | No data | No data |
| 191 | Yemen | | No data | No data | 42.00 | 55 | 72 | 66 | 88 | 85 |
| 192 | Zambia | | No data | No data | 53.00 | 61 | 94 | 93 | 69 | 82 |
| 193 | Zimbabwe | | No data | Data before 2007 | 54.00 | 63 | 83 | 82 | 56 | 92 |
